# Transcatheter Arterial Chemoembolization and Chemotherapy plus Sorafenib in a Large Hepatocellular Carcinoma with Arterioportal Shunt

**DOI:** 10.1155/2014/392403

**Published:** 2014-11-06

**Authors:** Jun Chen, Shixi Chen, Wei Xi, Bei Wu, Hui Yu, Yang Gao

**Affiliations:** Department of Radiology, Jiangsu Cancer Hospital and Cancer Hospital of Nanjing Medical University (NMU), 42 Baiziting Road, Nanjing 210009, China

## Abstract

*Introduction*. Arterioportal shunts (APS) are sometimes encountered in patients with hepatocellular carcinoma (HCC) and associated with poor prognosis. The management of HCC with APS is a challenge so far. *Case Presentations*. We report here in detail a 37-year-old man who was diagnosed as an advanced HCC accompanied with severe APS and treated by two sessions of transcatheter arterial chemoembolization (TACE) and three sessions of transcatheter arterial chemotherapy (TAC) plus sorafenib therapy. The tumor shrinks were revealed continuously during 152 days after the diagnosis. Although tumor progress emerged at 209 days after the diagnosis, the patient remarkably achieved 366-day survival. *Discussion*. TACE plus sorafenib may be a promising treatment for advanced HCC accompanied with APS. Prospective case-control studies should be advocated to evaluate the combination of TACE, TAC, and sorafenib in the management of HCC with APS.

## 1. Introduction

Arterioportal shunts (APS) are sometimes encountered in patients with hepatocellular carcinoma (HCC) and associated with poor prognosis. The management of HCC with APS is a challenge so far. Sorafenib and transcatheter arterial chemoembolization (TACE) combination shows promise as an effective and tolerable treatment strategy for intermediate stage and advanced HCC [1]. To our knowledge, no report concerning sorafenib and TACE combination in HCC with APS has been discussed in the English literatures. We report here in detail a case of large HCC with severe APS who achieved 366-day survival after TACE, transcatheter arterial chemotherapy (TAC), and sorafenib therapy.

## 2. Case Presentation

### 2.1. Case History

Written informed consent was obtained from the patient's wife for publication of this report and any accompanying images. A 37-year-old man presented with 2-month duration of intermittent discomfort in the right upper quadrant of the abdomen. His past medical history was inconsequential. His physical examination revealed no abnormality. This patient was referred for an ultrasound (US) examination for further evaluation which revealed a hypoechoic mass in the left lobe of the liver.

### 2.2. Laboratory Examination

Initial laboratory results upon presentation included serum alanine aminotransferase 58 U/L (normal level 0–40 U/L), aspartate aminotransferase 41 U/L (normal level 0–40 U/L), total bilirubin 13.7 *μ*mol/L (normal level 5.1–17.1 *μ*mol/L), conjugated bilirubin 5.6 *μ*mol/L (normal level 0–6 *μ*mol/L), albumin 41 g/L (normal level 35–55 g/L), *α*-fetoprotein (AFP) 6173 ng/mL (normal level 0–7 ng/mL), and cancer antigen 19-9 139.9 U/mL (normal level < 39 U/mL). Hepatitis B surface antigen, hepatitis B e-antibody, and hepatitis B core antibody were positive.

### 2.3. CT Findings

**Figure 1:**
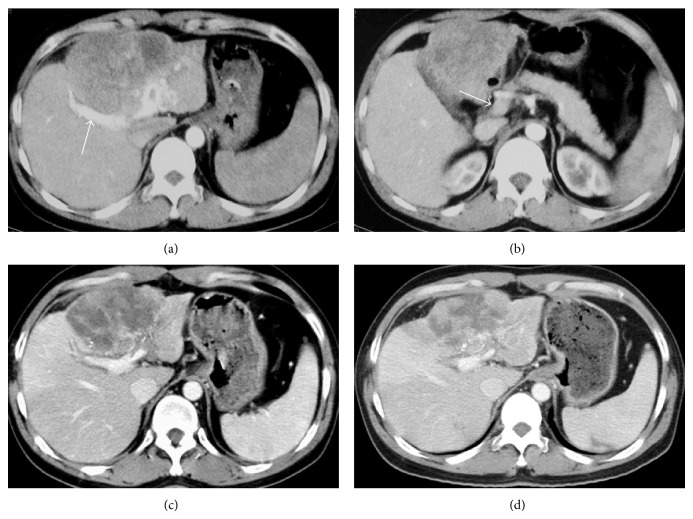
CT images. (a) and (b) refer to images at the initial presentation. (a) Shows a hypoattenuating mass with areas of heterogeneous enhancement in the left hepatic lobe. Visible right branch of portal vein was present in arterial phase axial CT image ((a), arrow), whereas main portal vein showed little contrast enhancement on arterial phase imaging ((b), arrow). (c) and (d) demonstrate the decreases of tumor diameter after the treatment at 59 and 152 days after the diagnosis, respectively.

Transverse unenhanced CT scan demonstrated a low-attenuation mass about 12.5 cm in diameter in the left hepatic lobe. Contrast-enhanced CT images confirmed a hypoattenuating mass with areas of heterogeneous enhancement (Figure 1(a)). Visible right branch of portal vein was present in arterial phase CT images, whereas main portal vein showed little contrast enhancement on arterial phase imaging (Figures 1(a) and 1(b)). CT images also identified a splenomegaly. Collectively, CT findings highly suggested a liver cancer with APS. Chest CT scan was not available at the initial presentation; however, chest X-ray examination of the patient revealed no abnormality.

### 2.4. Diagnosis and Treatment

**Figure 2:**
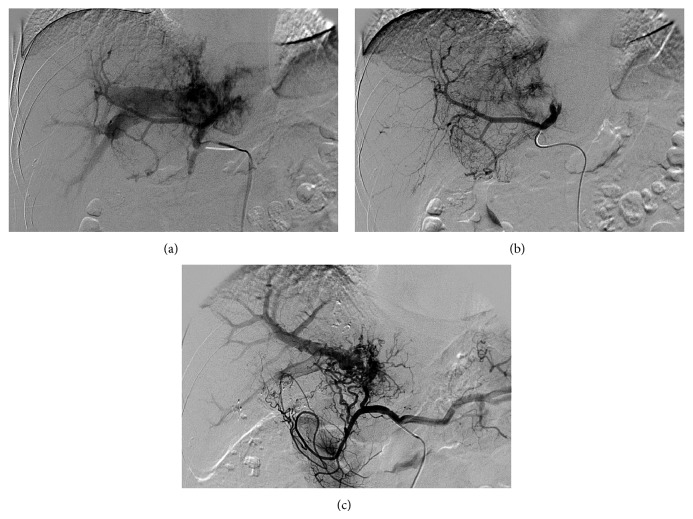
Hepatic arteriography images. Figures 1(a), 1(b), and 1(c) refer to 5, 5, and 151 days after the diagnosis of the tumor, respectively. Figure 1(a) shows early visualization of the right branch of portal vein which implies a prominent APS.  Figure 1(b) demonstrates the tumor staining and the occlusion of the APS due to the embolization with gelfoam through the left hepatic artery.  Figure 1(c) reveals reestablishment of the APS and tumor shrink after the treatment by TACE and TAC plus sorafenib.

According to clinical diagnosis and staging criteria in China [2], the mass reached a diagnosis as an advanced HCC (stage IIIa) based on a positive serum hepatitis B surface antigen, a high level of AFP, and a large mass with typical features of HCC in CT images. The patient was also evaluated as a stage IIIA (T_3_N_0_M_0_) HCC using the TNM staging system [3]. The patient preferred TACE to surgical resection, intravenous chemotherapy, and other therapeutic options. Five days after the diagnosis, angiography in the first session of TACE showed that the mass was supplied by the right and the left hepatic arteries (Figure 2(a)). An APS communication between the left hepatic artery and the right branch of portal vein was demonstrated by early visualization of the right branch of portal vein in hepatic arteriography (Figure 2(a)). The APS was successfully occluded by delivering gelfoam through the left hepatic artery (Figure 2(b)). An emulsion of 5 mL iodized oil and 40 mg doxorubicin, 1000 mg floxuridine, and 20 mg hydroxycamptothecin was then infused via the right hepatic artery. From the ninth day after the diagnosis, the patient orally took 400 mg sorafenib twice a day. In total, two sessions of TACE and three sessions of TAC were carried out for the treatment of the tumor during 209 days after the diagnosis (Table 1).

### 2.5. Following Up

Tumor shrinks were revealed by hepatic arteriography at 27 days, 84 days, and 151 days after the diagnosis of the tumor (Figure 2(c)). The diameter of the tumor in CT image was decreased continuously from 12.5 cm at the initial presentation to 8.8 cm at 151 days after the diagnosis (Figures 1(a), 1(c), and 1(d); Table 1). The present patient did not develop TACE or TAC induced adverse event. A moderate hand-foot skin reaction was noted as a sorafenib induced toxicity 27 days after the diagnosis of the tumor. About a month later, the patient was relieved from hand-foot skin reaction without dose reduction of sorafenib. Tumor progression was demonstrated by hepatic arteriography and CT images 209 days after the diagnosis. The patient has died from liver function failure at 366 days after the diagnosis of HCC. Table 1 shows the management and outcomes of the patient.

## 3. Discussion

Although TACE is a safe and effective treatment for HCC with major portal vein invasion, the reported median survival period was only 6.2 months [4]. Sorafenib, an oral multikinase inhibitor, has been shown to be effective and safe monotherapy in patients with advanced HCC [5]. The reported efficacy of a sorafenib and TACE combination appears to compare favorably with sorafenib or TACE monotherapies [1]. APS, a pathological communication between hepatic artery and portal vein, occurs sometimes in HCC patient when the tumor invades the portal vein system. To our knowledge, no study has reported the outcomes of HCC patients with APS after the treatment of TACE plus sorafenib. The present case reported an advanced HCC patient with severe APS in which the tumor shrinks were revealed continuously during 152 days after the diagnosis. Although tumor progress emerged at 209 days after the diagnosis, the patient remarkably achieved 366 days survival after TACE plus sorafenib therapy.

APS may impact on the management of TACE by the redistribution of arterial flow into portal venous flow in the patient with HCC; however, transcatheter arterial occlusion of APS is technically feasible [6]. Although technical successes were achieved immediately in the occlusion of APS using gelfoam through the left hepatic artery in the first and the second sessions of TACE, reestablishments of the APS were confirmed by hepatic arteriographies in the following sessions of TAC in the present case. Therefore, iodized oil and chemotherapy drugs were distributed into the tumors through the right hepatic artery in the first and the second sessions of TACE. In the following three sessions of TAC, only chemotherapy was performed through the proper hepatic artery.

AFP is probably useful in predicting prognosis and treatment response in HCC patients [7]. A study on localized concurrent chemoradiotherapy for advanced HCC indicated that outcomes were better in AFP responders than in AFP nonresponders [7]. The present patient showed an early decrease in AFP at 18 days after the diagnosis which is consistent with the tumor shrink identified by following hepatic arteriography and CT findings. Marked increase in AFP was revealed at 151 days after the diagnosis of HCC, but the diameter of the tumor decreased continuously during 152 days after the diagnosis in the present case. Shortly thereafter, the patient presented a tumor progression at 209 days after the diagnosis. AFP increasing may predict tumor progression and a relative poorer survival in patients with advanced HCC who receive sorafenib [8].

APS is considered as a contributor to the induction of portal vein hypertension and upper gastrointestinal bleeding. Although upper gastrointestinal bleeding did not occur in the present case, CT scanning at 335 days after the diagnosis did identify gastric varices that implied portal vein hypertension. Portal vein hypertension as well as potential peritoneal metastasis may cause the ascites of the patient revealed by CT images 213 days after the diagnosis.

In summary, the present case reported a large HCC with severe APS treated by TACE, TAC, and sorafenib. The patient achieved 366-day survival and died from liver function failure probably due to portal vein hypertension and tumor invasion of the whole liver. Prospective case-control studies should be advocated to evaluate the combination of TACE, TAC, and sorafenib in the management of HCC with APS.

## Figures and Tables

**Table 1:** Managements and outcomes.

Timing from diagnosis (d)	Management	Outcomes
0	CT laboratory	Tumor size: 12.5 × 8.5 cm AFP: 6173 ng/mL
5	TACE (gelfoam, an emulsion of 5 mL iodized oil and 40 mg doxorubicin, 1000 mg floxuridine, and 20 mg hydroxycamptothecin)	Angiography: tumor staining; APS
9	Sorafenib (400 mg twice a day)	Stopped at 354 days after the diagnosis
18	Laboratory	AFP: 4179 ng/mL
27	TACE (gelfoam, an emulsion of 5 mL iodized oil and 40 mg doxorubicin, 1000 mg floxuridine, and 20 mg hydroxycamptothecin)	Angiography: tumor shrink; reestablishment of the APS
59	CT	Tumor size: 9.6 × 7.1 cm
84	TAC (20 mg hydroxycamptothecin) laboratory	Angiography: tumor shrink; reestablishment of the APS; AFP: 4247 ng/mL
87	CT	Tumor size: 9.0 × 6.0
151	TAC (20 mg hydroxycamptothecin) laboratory	Angiography: tumor shrink AFP: 6281 ng/mL
152	CT	Tumor size: 8.8 × 6.0 cm
209	TAC (20 mg hydroxycamptothecin)	Angiography: tumor progression
213	CT	Tumor size: 10.7 × 7.1 cm; tumor metastasis in right hepatic lobe; mild ascites
262	CT	Tumor size: 10.9 × 7.4 cm; moderate ascites
335	CT	Tumor size: 14.4 × 8.4 cm; moderate ascites; gastric varices
354	CT	Tumor size: 14.4 × 8.5 cm; moderate ascites; gastric varices; both left and right hepatic lobes were invaded by the tumor
	laboratory	AFP: 80442 ng/mL
366	—	Died from liver function failure
